# Chiral nanostructures self-assembled from nitrocinnamic amide amphiphiles: substituent and solvent effects

**DOI:** 10.3762/bjnano.10.156

**Published:** 2019-08-05

**Authors:** Hejin Jiang, Huahua Fan, Yuqian Jiang, Li Zhang, Minghua Liu

**Affiliations:** 1 Beijing National Laboratory for Molecular Science (BNLMS), CAS Laboratory of Colloid, Interface and Chemical Thermodynamics, Institute of Chemistry, Chinese Academy of Sciences, Beijing 100190, China; 2 University of Chinese Academy of Sciences, Beijing 100049, China; 3 Laboratory for Nanosystem and Hierarchical Fabrication, CAS Center for Excellence in Nanoscience, National Center for Nanoscience and Technology, Beijing 100190, China

**Keywords:** chiral nanostructures, cinnamic acid, helicity inversion, nanoarchitectonics, self-assembly

## Abstract

Chiral nanostructures, such as α-helical proteins and double helix DNA, are widely found in biological systems and play a significant role in the biofunction of life. These structures are essentially fabricated through the covalent or noncovalent bonds between small chiral molecules. It is thus an important issue to understand how small chiral molecules can form chiral nanostructures. Here, using a series of isomeric nitrocinnamic amide derivatives, we have investigated the self-assembly behavior and the effect of the substituent position as well as the solvent on the formation of chiral nanostructures. It was found that totally different chiral nanostructures were formed due to the different positions of the nitro group on the cinnamic amide. Moreover, it was found that the chiral sense of the self-assembled nanostructures can be regulated by the solvent whereby helicity inversion was observed. This work provides a simple way to regulate the self-assembly pathway via molecular design and choice of solvent for the controlled creation of chiral nanostructures.

## Introduction

The helical structure is widely found in biological systems and is considered to be a basic characteristic of living matter and perhaps even a requirement for life [1–2]. For example, the α-helix of peptides, the DNA double helix, and the triple helix of collagens are vital biological structures. It is an important issue to understand how such chiral nanostructures can be formed from simple small molecules. Nanoarchitectonics is a useful technology to create a new class of materials by controlled arrangement of structural nanoscale units such as atoms, molecules and assemblies [3–5]. It is also an efficient strategy to mimic helical structures [6–8]. Based on the concept of architectonics, amino acids [9–11], oligopeptides [12–13], saccharides [14–16], steroids [17–18] and diaminocyclohexane derivatives [19–20] have been reported to self-assemble into helical structures, mimicking the natural helical structures found in biological systems. Generally speaking, the common feature of these building blocks is that chiral centers are contained. The synergy between various noncovalent interactions, including hydrogen bonding [21–22], π–π stacking [23–24], and hydrophobic interactions [25–26] provided by other moieties in self-assembly units, cause the chiral information to be accumulated and finally to express as helical structures. Then the question arises: will the chiral centers absolutely determine the chiral sense of the formed structures? Or do other noncovalent interactions have an influence on the chiral structures? Isomers with the same chiral center are good model compounds to investigate the effect of molecular structure on the chiral sense of self-assembled structures. In our previous study [27], three isomeric pyridine-containing ʟ-glutamic amphiphiles have been found to self-assemble into different nanostructures including nanofibers, nanotwists and nanotubes, depending on the substituent position in the pyridine ring. However, we did not observe inversion in the helical sense of the formed self-assembled nanostructures due to the macroscopic chirality of nanofibers and nanotubes, which makes them difficult to be directly detected by a microscope.

On the other hand, helical architectures in many bimolecular systems have been shown to exhibit helicity inversion along with specific biofunctional transformations upon stimuli [28]. Thus, many attempts have been made towards understanding the reversal of handedness of helical biological systems. The chiral self-assembly gained from various noncovalent interactions is a very good biomimetic system due to the intrinsic dynamic nature of such materials and smart response to external stimuli. There are some works on the dynamic helical inversion in self-assembled structures triggered by the change of pH value [29–30], solvents [31–32], temperature [33–34], and photo-irradiation [35–36]. Inverse chiral nanostructures have exhibited their tunable functions in the field of asymmetric catalysts [37–39], chiral separation [40–41], and circular polarized luminescence [42–43]. In this case, tunable chiral functions can be found in the compounds with the same absolute configuration depending on the environmental conditions. Thus, more and more efforts should be made towards exploring self-assembled structures demonstrating helicity inversion, especially when the inversion directly occurs in nanostructures, i.e., chiral nanostructures with left-handed (right-handed) sense changed to right-handed (left-handed) upon external stimuli.

Based on these considerations, herein, we design three isomeric nitrocinnamic amide-containing ʟ-glutamic amphiphiles, which differ in the position of the nitro group on the cinnamic amide, and interestingly, we found that chiral structures with totally opposite helical sense can be obtained in the self-assembly of these ʟ-glutamic amphiphiles, depending on the position of the nitro group. Furthermore, according to our previous study [43], the cinnamic amide assembly was closely related to the choice of solvent, and the photo-dimerization of the cinnamic amide moiety only occurred for methanol and ethanol. Other solvents could not be shown to induce this kind of transformation. We speculated that methanol or ethanol may affect the hydrogen bonding between the amide moieties, which differed from other kinds of solvents. In order to further confirm the specificity of methanol and investigate whether the solvent can cause the helicity inversion, in this study, we explore the self-assembly behavior of three nitrocinnamic amide-containing ʟ-glutamic lipids in various solvents.

## Results and Discussion

### Self-assembly of NCLG

**Figure 1:**
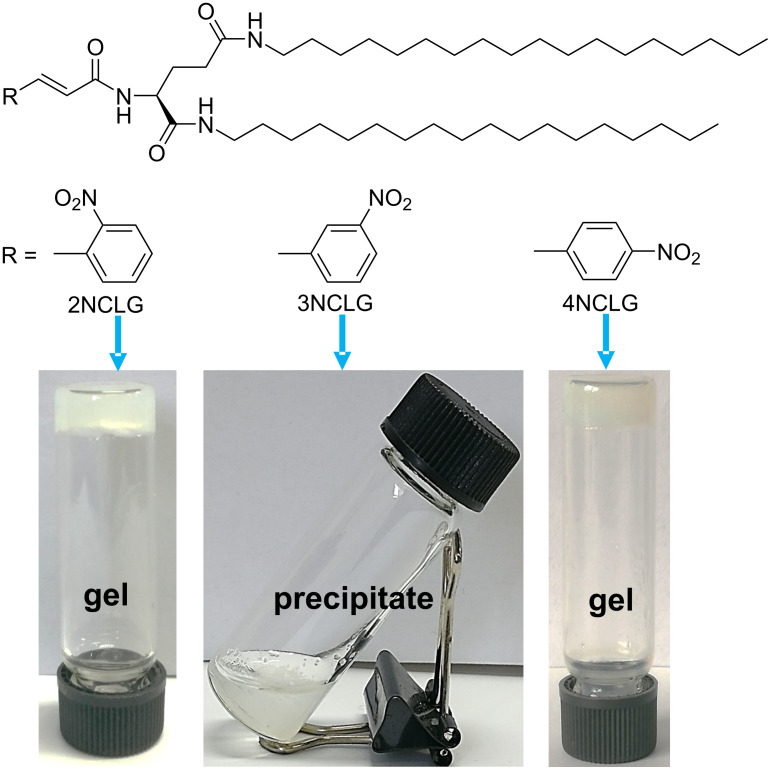
Molecular structure of three nitrocinnamic amide-containing ʟ-glutamic amphiphiles and photographs of their self-assembled molecules in ethanol.

Three chiral amphiphile materials, named as 2NCLG, 3NCLG and 4NCLG (as an acronym related to the precursor nitrocinnamic ʟ-glutamic acid (NCLG)), were designed and synthesized by covalently linking three *trans*-nitrocinnamic acids (2-NCA, 3-NCA and 4-NCA), respectively, to the organic lipid gelator *N*,*N*’-bis(octadecyl)-ʟ-glutamic diamide (LGAm) (as shown in Figure 1). The difference between the three gelators is the substituent position of the nitro group on cinnamic acid. All of these gelators could be dissolved in organic solvents with heating, and the self-assembled molecules formed after cooling down to ambient temperature. At the same concentration (12 mg/mL), 2NCLG and 4NCLG formed white gels in EtOH, while 3NCLG precipitated in EtOH, as shown in Figure 1.

### SEM characterization

**Figure 2:**
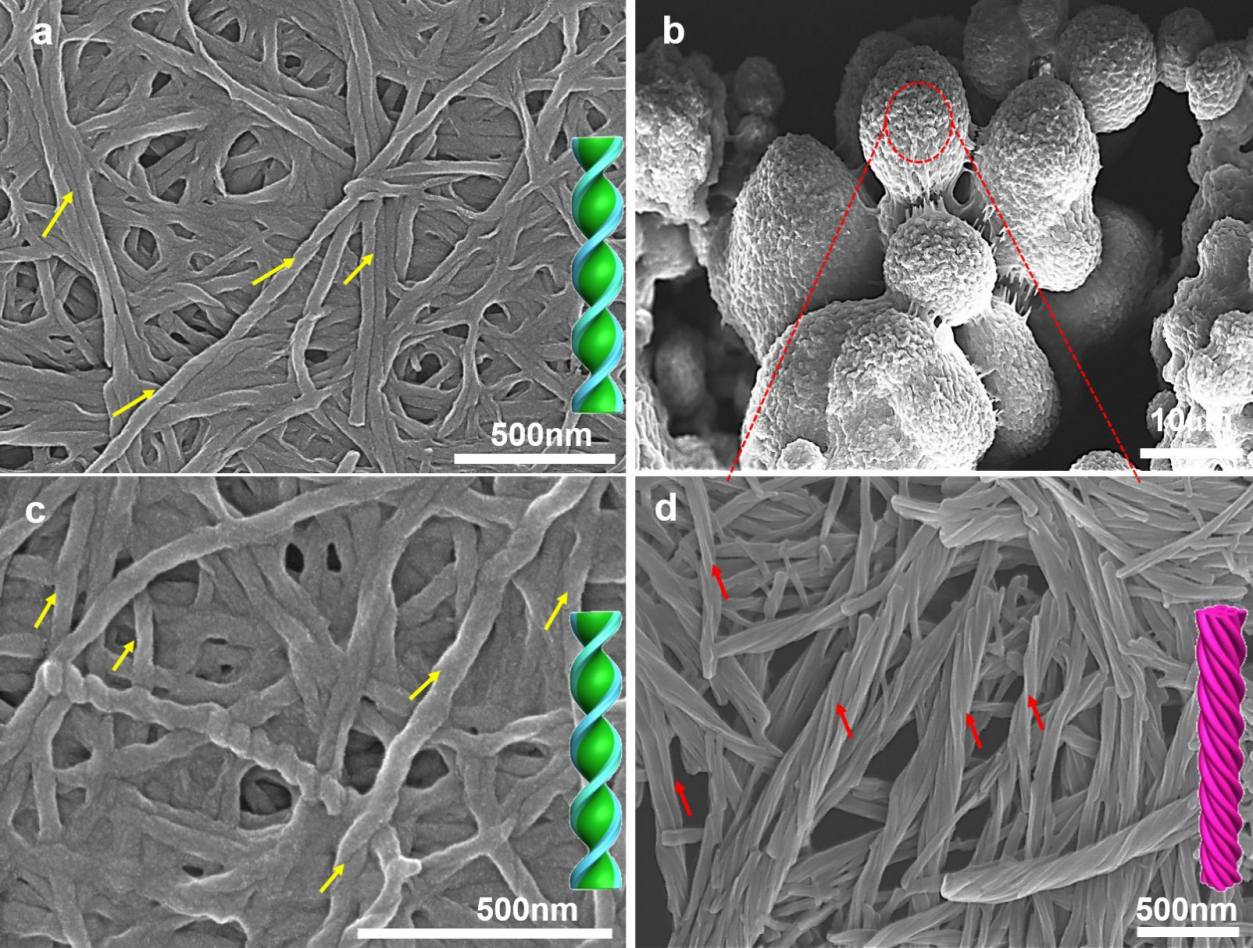
SEM images of NCLG assemblies in EtOH: (a) 2NCLG, (b,d) 3NCLG, and (c) 4NCLG self-assembled structures. The concentration is 12 mg/mL.

Furthermore, the morphology of the 2NCLG, 3NCLG and 4NCLG assemblies in ethanol was analyzed by scanning electron microscopy (SEM). Figure 2 shows the detailed SEM images of the self-assembled structures. Upon SEM observation, 2NCLG self-assembled into a right-handed helical nanofiber with a helical pitch of about 250 nm and a width of approximately 70 nm, as shown in Figure 2a. As for 4NCLG assemblies, a similar right-handed helical nanofiber was obtained (Figure 2c). In contrast, a left-handed superhelical structure with a helical pitch of around 500 nm was observed in the 3NCLG system, which was formed by dozens of nanofibers. The nanohelix finally aggregated into microspherical structures (Figure 2b,d). Because of the wide field of view of the SEM illumination over the 3NCLG (Supporting Information File 1, Figure S1), the process of self-assembly was fast and the formed nanofiber structures tangled together into a superhelix. The superhelix then bundled together and formed microspherical structures. The microspherical structures finally aggregated together and precipitated from the EtOH solvent. However, for the 2NCLG and 4NCLG structures, the process of self-assembly was slower than for 3NCLG and the nanofiber entangled together and formed 3D network gels. The SEM results reveal that the nanoscale chirality of the 3NCLG assembly is opposite to that of the 2NCLG and 4NCLG assemblies. It is suggested that the nanoscale chirality of the formed nanostructures did not strictly follow the chirality of the chiral carbon centers in glutamide. We speculate that the substituent position of NO_2_ might affect the arrangement of molecules in the self-assembly process and subsequently lead to a different packing model of the NCLG compounds.

### UV–vis and circular dichroism spectra

**Figure 3:**
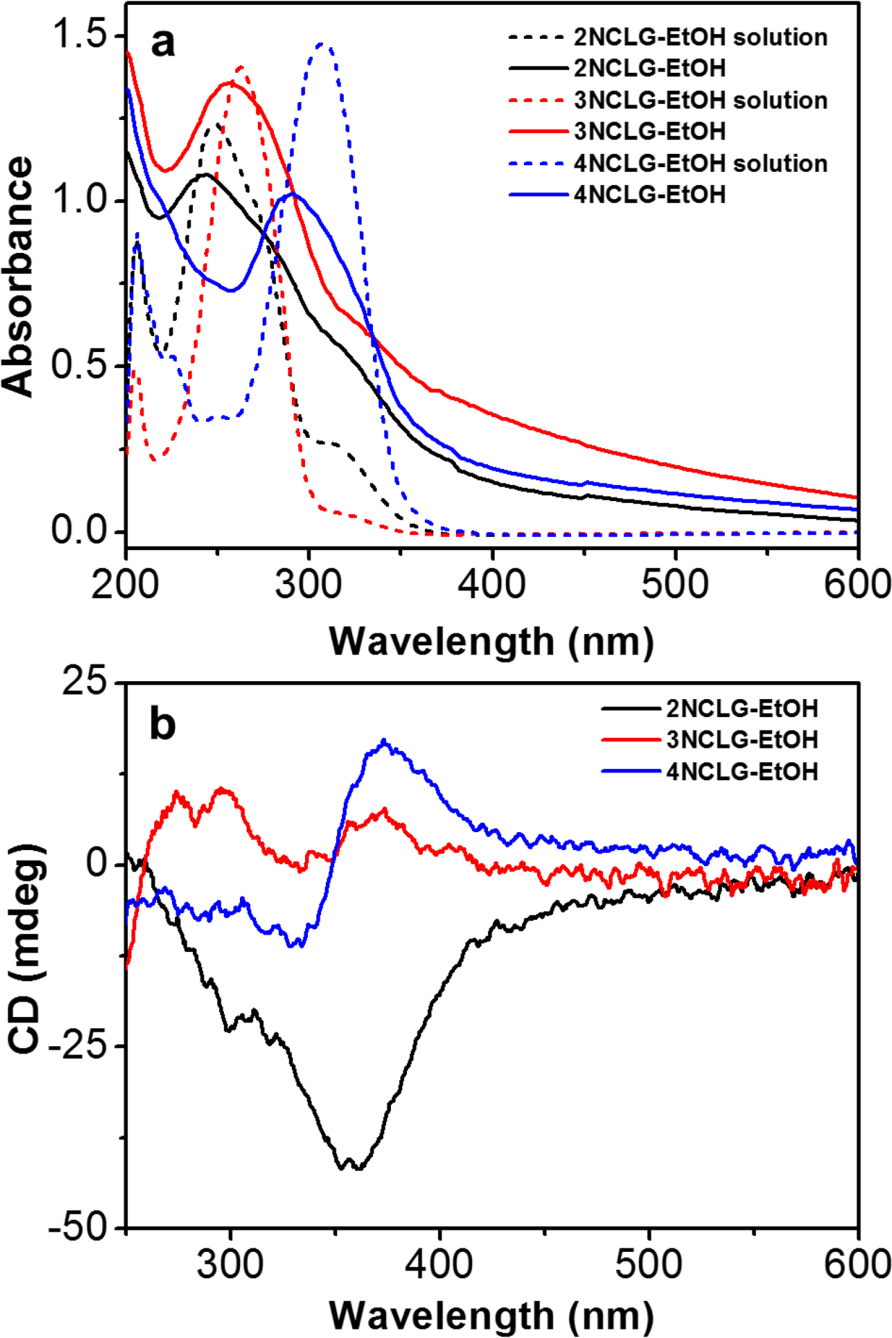
(a) UV–vis spectra of 2NCLG, 3NCLG, 4NCLG ethanol solutions and self-assembled molecules. (b) CD spectra of 2NCLG, 3NCLG and 4NCLG self-assembled molecules.

In order to further understand the different self-assembly behaviors among the NCLG molecules, the UV–vis spectra and circular dichroism (CD) spectra were investigated (Figure 3). Figure 3a shows the UV–vis spectra of NCLG solutions and assemblies in ethanol. It can be clearly observed that the 2NCLG, 3NCLG and 4NCLG solutions exhibited main absorption bands at approximately 249 nm, 263 nm and 306 nm, respectively, which can be ascribed to π–π* transitions. In addition, 2NCLG and 3NCLG showed a shoulder absorption peak at approximately 315 and 325 nm, respectively, while all the main absorption bands of the NCLG assemblies in ethanol showed a blue shift to 241 nm, 258 nm and 293 nm, respectively. This result suggests a H-like aggregation of NCLG molecules through π–π stacking. CD spectroscopy is considered to be a useful technique to monitor the supramolecular assembly. Consequently, distinct CD signals were obtained for the assemblies of the three NCLG molecules, as shown in Figure 3b. A negative Cotton effect at around 355 nm was observed for the 2NCLG gel, while a positive Cotton effect at about 300 nm and 370 nm appeared for the 3NCLG assembly. As for the 4NCLG system, a positive Cotton effect was detected at 371 nm and a negative one at 333 nm with a crossover at 348 nm. These CD bands were wider than the absorption bands of NCLG assemblies, which may be due to the chiral scattering [44]. Similar to previous reports [45], the hot solution of the three NGLG molecules was CD silent, while the distinct CD signals of the gels and precipitates supported the theory that the formation of assemblies and the chirality of ʟ-glutamic acid was transferred to the cinnamic amide moiety. In our previous work, the self-assembly of cinnamic acid derivatives was photo-responsive, while in this work, we found that the self-assembled molecules of the three gelators did not show photo-responsive properties under UV-light irradiation, in the CD spectra or in the morphology of the nanostructures. Supporting Information File 1, Figure S2 shows that the morphology of all the nanostructures remained intact, and the supramolecular chirality of the self-assembled molecules monitored by CD did not show inversion.

### X-ray diffraction analysis

**Figure 4:**
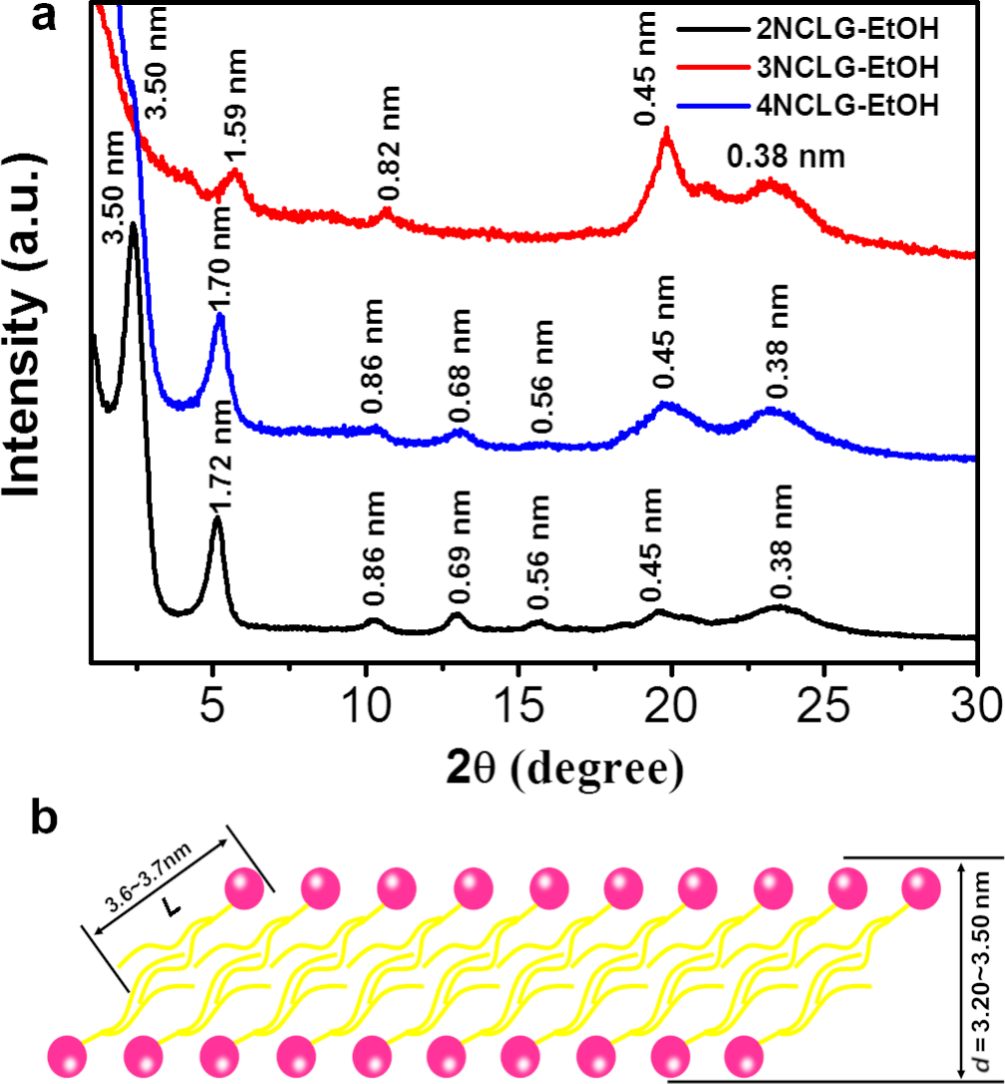
(a) XRD patterns of the 2NCLG assembly (black), 3NCLG assembly (red) and 4NCLG assembly (blue). (b) Proposed packing model of the three molecules.

To understand the different structure of the three NCLG compounds, X-ray diffraction (XRD) measurements were further adopted to evaluate the assembled structures of the three gelators. As shown in Figure 4a, for 2NCLG xerogels, a series of sharp diffraction peaks were observed at 2θ = 2.51, 5.11, 10.21, 12.83 and 15.57, with a *d*-spacing ratio of 1:1/2:1/4:1/5:1/6. The diffraction pattern is related to the lamellar structure with the *d*-space of 3.50 nm. As for 4NCLG gels, the XRD pattern was almost similar to the 2NCLG assembly. A number of diffraction peaks occurred at 2θ = 2.51(100), 5.20(200), 10.23(400), 12.97(500) and 15.59(600), which clearly illustrated that the 4NCLG assembly also presented a lamellar structure. However, only 2θ = 5.56 and 10.74 diffraction peaks were observed for the 3NCLG assembly. Considering the structural similarity of the three NCLG molecules, we speculated that the first diffraction peak for 3NCLG did not appear. Then the lamellar structure with a *d*-spacing of ≈3.20 nm was obtained for the 3NCLG assemblies, although the order is lower than that of 2NCLG and 4NCLG. Actually, the length (*L*) of the NCLG compounds is about 3.6–3.7 nm, as simulated by gaussview. The XRD pattern revealed that the *d*-spacing of the lamellar structure was 3.50 nm for 2NCLG and 4NCLG and 3.20 nm for 3NCLG, which is shorter than the length of two molecules (actually, even less than one molecular length) (Figure 4b). This result indicates that the NCLG assemblies might form a bilayer structure with high interdigitation of the alkyl chains, where the bilayer structure experiences a large tilt.

### Fourier-transform infrared (FTIR) spectra

**Figure 5:**
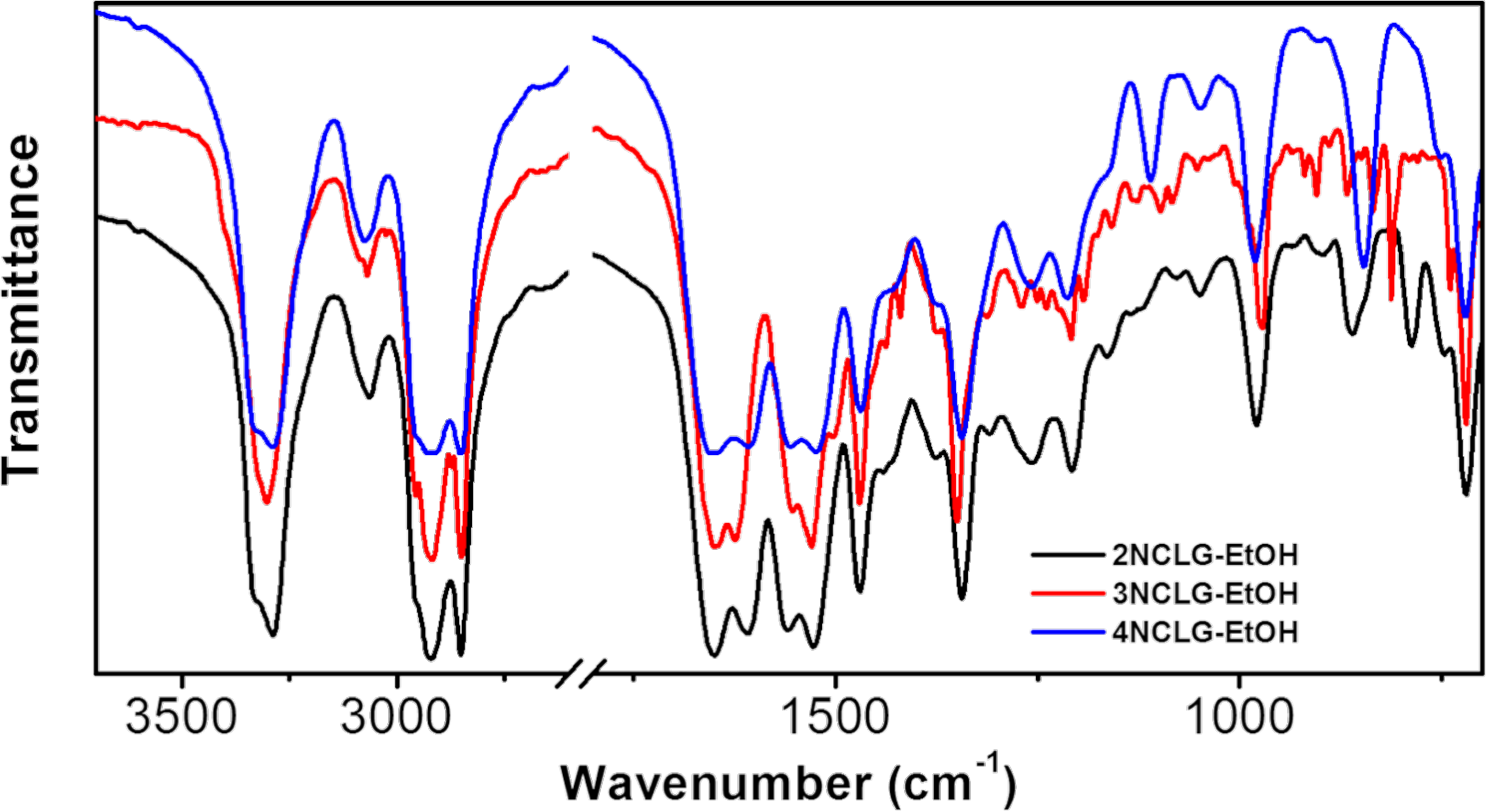
FTIR spectra of the 2NCLG assembly (black), 3NCLG assembly (red) and 4NCLG assembly (blue) obtained in EtOH.

In order to elucidate the formation mechanism of the helicity and nanostructures of the self-assembled molecules, FTIR spectroscopy was employed to evaluate the formation mechanism of self-assembly. As shown in Figure 5, for the 2NCLG and 4NCLG assemblies, two absorption bands at ≈3330 cm^−1^ and ≈3284 cm^−1^ were observed, which can be ascribed to the N–H stretching vibration. While for 3NCLG, the shoulder absorption band showed a red shift to ≈3328 cm^−1^ and the main absorption band displayed a blue shift to ≈3302 cm^−1^, which illustrated the weaker hydrogen bonding between 3NCLG molecules than that of 2NCLG and 4NCLG. The CH_3_ and CH_2_ stretching vibration bands of alkyl chains at ≈2955, 2920 and 2849 cm^−1^ showed no obvious change. The band at ≈1650 cm^−1^ was almost the same for all the three assemblies, which was assigned to the C=O stretching vibration of the amide I. However, the amide II band of the C–N–H bending vibration of the 2NCLG and 4NCLG assemblies was at ≈1560 cm^−1^, while the band red-shifted to ≈1554 cm^−1^ for the 3NCLG assemblies. It also indicated that the hydrogen bonding between 3NCLG was weaker than the other two compounds. In addition, the absorption bands at ≈1520–1530 cm^−1^ and ≈1340–1350 cm^−1^ could be ascribed to the antisymmetric and symmetric stretching vibration of the nitro group, respectively. The absorption bands at ≈970–980 cm^−1^ were assigned to *trans*-vinylene C–H out-of-plane deformations and the ≈779–785 cm^−1^ absorption bands were attributed to *cis*-vinylenene C–H out-of-plane deformations. The detailed information of the FTIR spectra is given in Table 1.

**Table 1:** Assignment and description of FTIR absorption bands of the three NCLG assemblies.

Frequency/cm^−1^			Assignment and description
2NCLG	3NCLG	4NCLG	

3330	3328	3330	N–H stretching vibration
3284	3298	3284	N–H stretching vibration
2957	2955	2955	CH_3_ asymmetric stretching vibration
2920	2920	2920	CH_2_ asymmetric stretching vibration
2851	2849	2849	CH_2_ symmetric stretching vibration
1651	1642	1650	amide I band (C–O) stretching vibration
1608	1610	1608	C=O stretching vibration in benzene ring
1561	1588	1559	amide II band (C–N–H) stretching vibration
1525	1549	1524	NO_2_ antisymmetric stretch vibration
1344	1325	1344	NO_2_ symmetric stretch vibration
979	971	979	*trans*-vinylene C–H out-of-plane deformation
785	779	none	*cis*-vinylene C–H out-of-plane deformation

Based on the data of FTIR spectra, we speculate that the helicity inversion of 3NCLG nanostructures might be due to the weak hydrogen bonding between 3NCLG molecules as compared to that of 2NCLG and 4NCLG. It also caused a relatively loose molecular packing of 3NCLG, which was also illustrated in the XRD patterns.

**Figure 6:**
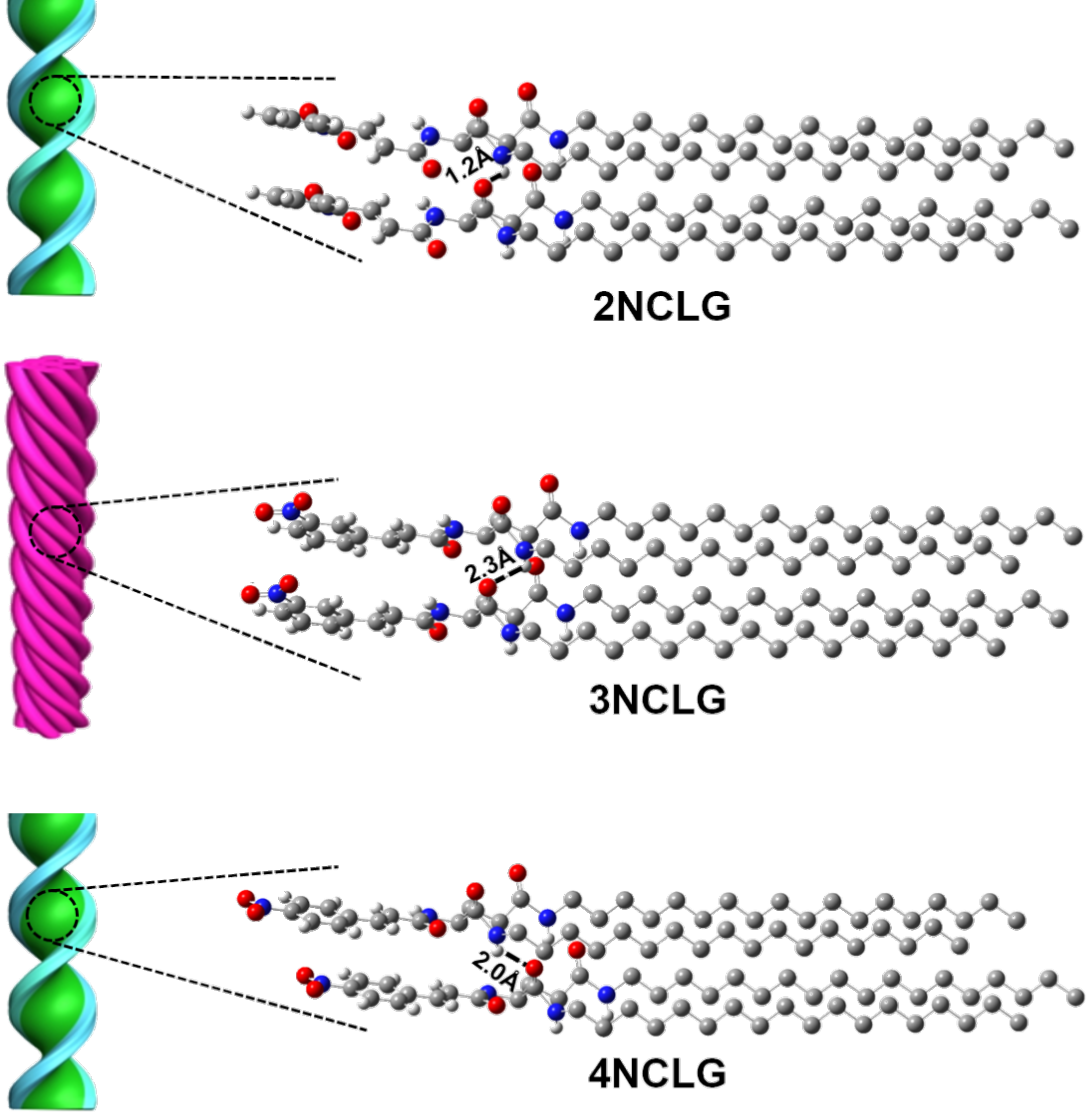
Illustration on the self-assembly mechanism of NCLG isomers.

Next, we tried to simulate the packing model of the three NCLG compounds and two randomly adjacent molecules of NCLG were extracted from their crystals. As shown in Figure 6, it can be clearly observed that the packing model of the 2NCLG molecules was very similar to that of the 4NCLG molecules. Both of the molecules are misaligned in their crystal, which indicates that the bottom molecule is not directly below the upper one. Additionally, the length of intermolecular hydrogen bonds of 2NCLG and 4NCLG assemblies were found to be 1.2 Å and 2.0 Å, respectively. While for 3NCLG, the bottom molecule is right below the upper one and the length of intermolecular hydrogen bonds is 2.3 Å (i.e., longer than that of 2NCLG and 3NCLG). This result further demonstrated that the hydrogen bonding of the 3NCLG assembly was weaker than for the 2NCLG and 4NCLG assemblies. The difference in hydrogen bonding eventually led to different packing of the self-assembled molecules. The strong hydrogen bonding favored the formation of right-handed nanohelical structures, while the opposite chirality of the left-handed superhelix of 3NCLG was attributed to the weak hydrogen bonding in these assemblies.

### Helicity inversion in self-assembly: effect of solvent

**Figure 7:**
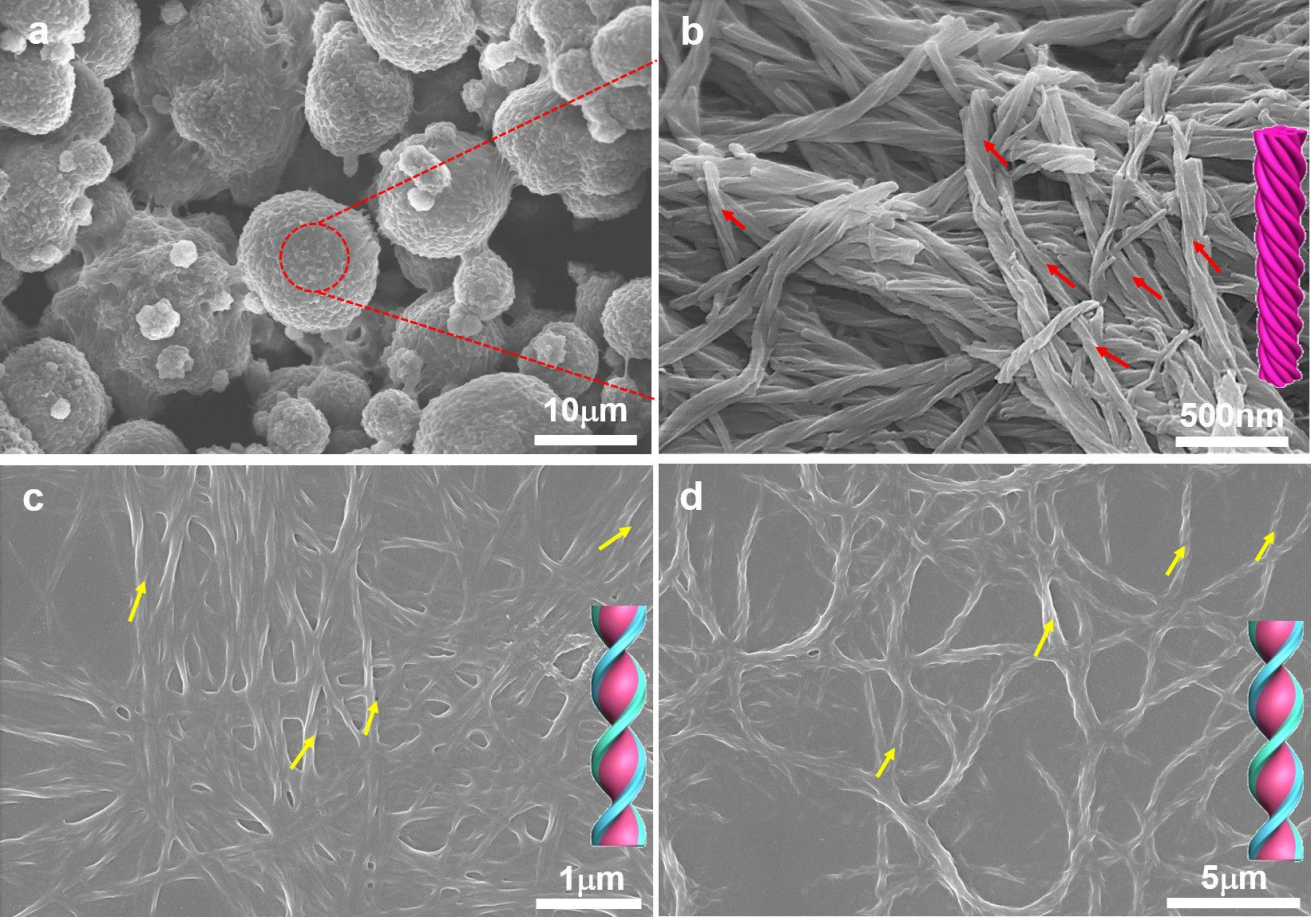
SEM images of the 3NCLG assembly in (a,b) MeOH, (c) DMF, and (d) THF. The concentration is 12 mg/mL.

In addition, the effect of solvent on the 3NCLG self-assembly was also explored. The 3NCLG molecule could readily form transparent gels in DMF and THF when the concentration was above 8 mg/mL, while it formed a precipitate in methanol at the same concentration, likely indicating the different self-assembly behaviors of 3NCLG. Firstly, SEM was used to characterize the xerogels and dried precipitate, as shown in Figure 7. As we expected, the left-handed superhelix of 3NCLG precipitated in methanol was observed and the nanohelix (Figure 7a) further aggregated into microspherical structures (Figure 7b), which was likely due to the nanostructure of the ethanol assembly. However, both DMF organogels and THF organogels consist of right-handed helical nanostructures (Figure 7c and 7d). We speculated that the opposite chirality in DMF and THF assemblies to that of those in ethanol and methanol was also related to the intermolecular hydrogen bonding. Besides, 2NCLG formed right-handed nanohelix both in DMF and THF, which is the same as in ethanol. The 4NCLG gelator formed nanotube structures both in DMF and THF, which is different from the nanohelix that formed in ethanol (Supporting Information File 1, Figure S3). These results indicated that the choice of solvent had a significant effect on the formed nanostructures.

**Figure 8:**
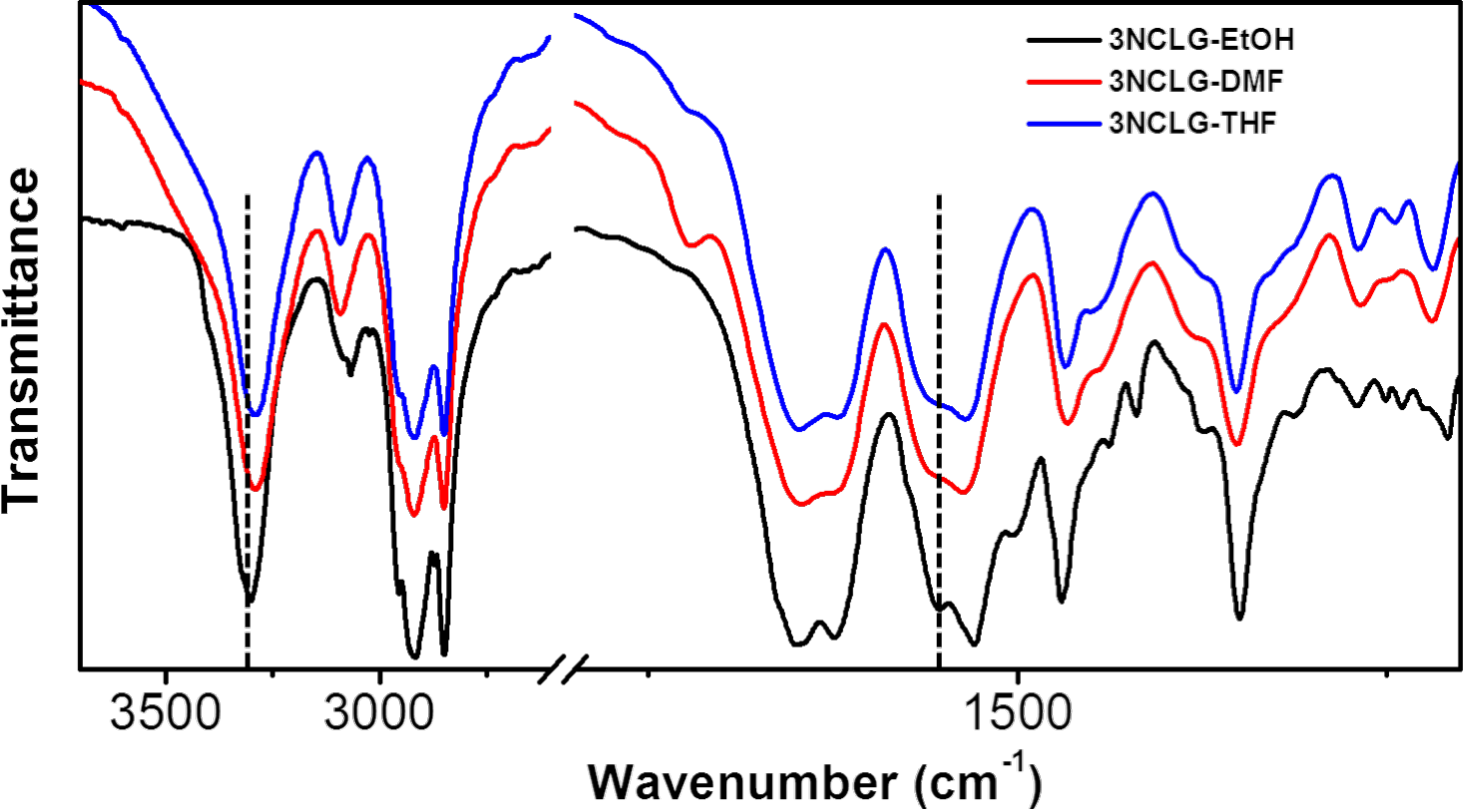
FTIR spectra of the 3NCLG assembly in ethanol (black), in DMF (red) and in THF (blue).

To support our speculation, the 3NCLG assemblies in DMF and THF were monitored by FTIR spectroscopy, as shown in Figure 8. We mainly focused on the N–H stretching vibration, the amide I stretching vibration and amide II bending vibration. For 3NCLG assemblies obtained in DMF and THF, the main absorption bands of the N–H stretching vibration were observed at ≈3292 cm^−1^ which showed a red shift from ≈3328 cm^−1^ compared to the N–H band of 3NCLG in ethanol. This result illustrates the stronger hydrogen bonding in 3NCLG DMF and THF assemblies. Moreover, the amide II, C–N–H bending vibration blue-shifted to ≈1562 cm^−1^ for 3NCLG in DMF and THF compared to 3NCLG in ethanol assemblies, which also proved that a relatively strong hydrogen bonding exists in the 3NCLG assemblies formed in DMF and THF. This may be because the protic solvents ethanol and methanol could affect the hydrogen bonding between 3NCLG molecules. The result is that the 3NCLG self-assembled molecules obtained in ethanol and methanol present opposite helicity to those obtained in DMF and THF.

## Conclusion

In conclusion, we found that the self-assembled structures of three isomeric nitrocinnamic amide derivatives showed variable helical sense depending on the substituted position of the nitro group of the cinnamic amide. This varying helical sense occurred even though the molecular chirality of the three NCLG molecules was derived from the same source, i.e., ʟ-glutamic acid. At the same time, the variation in the substituted position also led to different gelation abilities. Additionally, it was demonstrated that the chirality of a nanostructure can also be regulated by choice of solvents. The chiral inversion of these nanostructures was found to be related to the intermolecular hydrogen bonding of cinnamic amide amphiphiles.

## Experimental

### Chemicals and materials

*N*-(*tert*-Butoxycarbonyl)-ʟ-glutamic acid (Boc-ʟ-Glu) and 4-nitrocinnamic acid (4-NCA) were purchased from TCI. 1-Octadecylamine was bought from Alfa Aesar. 1-Hydroxybenzotriazole (HOBt) was purchased from dams-beta. *Trans*-2-nitrocinnamic acid (2-NCA), *trans*-3-nitrocinnamic acid (3-NCA) and (*N*-(3-dimethylaminopropyl)-*N*'-ethylcarbodiimide hydrochloride (EDC·HCl) were purchased from J&K. Dichloromethane, sodium bicarbonate (NaHCO_3_) and hydrochloric (HCl) and were supplied by Beijing Chemical Regent Company (China). Ethanol, *N*,*N*-dimethylformamide and tetrahydrofuran were bought from Xilong Scientific. Milli-Q water (18.2 MΩ·cm) was used in all cases. All the chemicals and solvents were bought from commercial suppliers and used without further purification.

### Synthesis of NCLG gelators

**Scheme 1:**
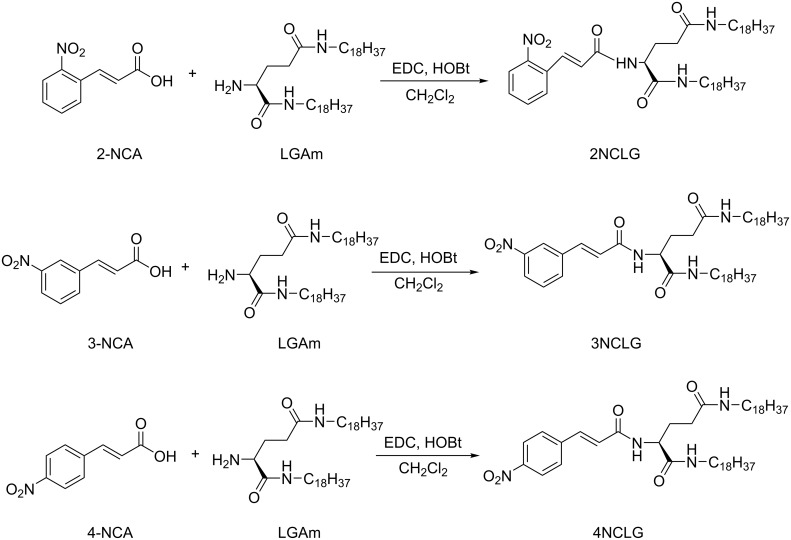
Synthesis scheme of the target chiral compounds 2NCLG, 3NCLG and 4NCLG.

The synthesis and characterization of the precursors *N*,*N*’-bisoctadecyl-ʟ-glutamic diamide (LGAm) has been reported previously [46]. 2-NCA, 3-NCA and 4-NCA (0.59 g, 3.07 mmol) were respectively dispersed into 200 mL of dichloromethane with *N*,*N*'-bisoctadecyl-ʟ-glutamine (LGAm; 1.0 g, 1.54 mmol). The mixture was then stirred for 30 min. Then, 1-hydroxybenzotriazole (HOBt; 0.42 g, 3.07 mmol) and *N*-(3-dimethylaminopropyl)-*N*'-ethylcarbodiimide hydrochloride (EDC·HCl; 0.59 g, 3.07 mmol) were added to the reaction flask. The mixture was then stirred and heated under reflux for 3 days. The solvent was removed by filtration and the residue was washed with dichloromethane several times. The crude products were then heated to dissolve in ethanol (50 mL) and added into nearly saturated aqueous NaHCO_3_ solution (500 mL) with stirring for 20 min. The sovent was then removed by filtration and the white product was washed with water. The dried product was dissolved in ethanol (50 mL) again by heating and the hot solution was then poured into aqueous HCl solution (500 mL). Finally, the dried product was purified by recrystallization four times in EtOH/THF to obtain the target compounds: 2NCLG (0.71 g, 56% yield), 3NCLG (0.94 g, 74% yield) and 4NCLG (0.90 g, 71% yield) (Scheme 1).

**2NCLG:** ^1^H NMR (500 MHz, DMSO-*d*_6_, 100 °C, TMS) δ 0.83–0.94 (t, 6H), 1.20–1.50 (m, 60H), 1.36–1.50 (m, 4H), 1.80–1.90 (m, 1H), 1.93–2.00 (m, 1H), 2.10–2.20 (m, 2H), 3.12–3.17 (m, 4H), 4.33–4.44 (q, 1H), 6.73–6.82 (d, 1H), 7.35–7.43 (s, 1H), 7.53–7.66 (m, 2H), 7.68–7.81 (m, 3H), 7.96–8.07 (m, 2H); MALDI–TOF–MS *m*/*z*: [M]^+^ calcd. for C_50_H_88_N_4_O_5_, 825.26; found, [M + Li]^+^ 833.5, [M + Na]^+^ 847.5.

**3NCLG:** ^1^H NMR (500 MHz, DMSO-*d*_6_, 100 °C, TMS) δ 0.83–0.93 (t, 6H), 1.19–1.50 (m, 60H), 1.36–1.50 (m, 4H), 1.78–1.90 (m, 1H), 1.93–2.04 (m, 1H), 2.10–2.19 (m, 2H), 3.03–3.17 (m, 4H), 4.34–4.44 (q, 1H), 6.90–7.00 (d, 1H), 7.34–7.44 (s, 1H), 7.50–7.60 (m, 2H), 7.62–7.74 (d, 1H), 7.90–8.02 (m, 2H), 8.14–8.22 (m, 1H), 8.34–8.40 (s, 1H); MALDI–TOF–MS *m*/*z*: [M]^+^ calcd. for C_50_H_88_N_4_O_5_, 825.26 [M]^+^; found, [M + Li]^+^ 833.5, [M + Na]^+^ 847.5.

**4NCLG:** ^1^H NMR (500 MHz, DMSO-*d*_6_, 100 °C, TMS) δ 0.80–0.93 (t, 6H), 1.16–1.50 (m, 60H), 1.36–1.52 (m, 4H), 1.81–1.93 (m, 1H), 1.93–2.04 (m, 1H), 2.09–2.29 (m, 2H), 3.03–3.16 (m, 4H), 4.33–4.43 (q, 1H), 6.88–6.98 (d, 1H), 7.33–7.45 (s, 1H), 7.48–7.62 (m, 2H), 7.76–7.85 (d, 2H), 7.95–8.07 (d, 1H), 8.17–8.28 (d, 2H); (MALDI–TOF–MS) *m*/*z*: [M]^+^ calcd. for C_50_H_88_N_4_O_5_, 825.26; found, [M + Li]^+^ 833.5, [M + Na]^+^ 847.5.

### General characterization

MALDI–TOF–MS was recorded on a Bruker Autoflex III instrument. Nuclear magnetic resonance (NMR) was characterized on a Bruker AVANCE III HD 500 machine. The gel and precipitate were cast onto single-crystal silica plates and then coated with a thin layer of Pt after drying to increase the contrast. After that, the morphology was observed with a Hitachi S-4800 FE-SEM operating at an accelerating voltage of 10 kV. UV–vis spectra were recorded with a Hitachi U-3900 spectrophotometer in quartz cuvettes (light path 0.1 mm and 1 cm). CD spectra were measured with a JASCO J-810 CD spectrophotometer in quartz cuvettes with a 0.1 mm path length over a range of 200–800 nm. XRD analysis was performed on a Rigaku D/Max-2500 X-ray diffractometer (Japan) with Cu Kα radiation (λ =1.5406 Å). The operating voltage was 40 kV and a current of 200 mA was used. The samples were cast on silicon substrates and dried in air for XRD measurements. Fourier-transform infrared (FTIR) spectroscopy was recorded with a Bruker TENSOR-27 spectrophotometer. The testing range was 400–4000 cm^−1^ and the wavenumber resolution was 4 cm^−1^ at room temperature.

## Supporting Information

File 1 Additional experimental data.
