# Supplementary material for: Chiral nanostructures self-assembled from nitrocinnamic amide amphiphiles: substituent and solvent effects
Source: Beilstein J Nanotechnol. 2019 Aug 5;10:1608–17. doi: 10.3762/bjnano.10.156 (PMC6693415; doi:10.3762/bjnano.10.156)
Supplement: File 1 — Additional experimental data. [file Beilstein_J_Nanotechnol-10-1608-s001.pdf]

BEILSTEIN JOURNAL OF NANOTECHNOLOGY

Supporting Information

for

Chiral nanostructures self-assembled from nitrocinnamic amide amphiphiles: substituent and solvent effects

Hejin Jiang, Huahua Fan, Yuqian Jiang, Li Zhang and Minghua Liu

Beilstein J. Nanotechnol. **2019,** *10,* 1608–1617. doi:10.3762/bjnano.10.156

Additional experimental data

License and Terms: This is a supporting information file under the terms of the Creative Commons Attribution License (http://creativecommons.org/licenses/by/4.0). Please note that the reuse, redistribution and reproduction in particular requires that the authors and source are credited.
The license is subject to the *Beilstein Journal of Nanotechnology* terms and conditions: (https://www.beilstein-journals.org/bjnano)

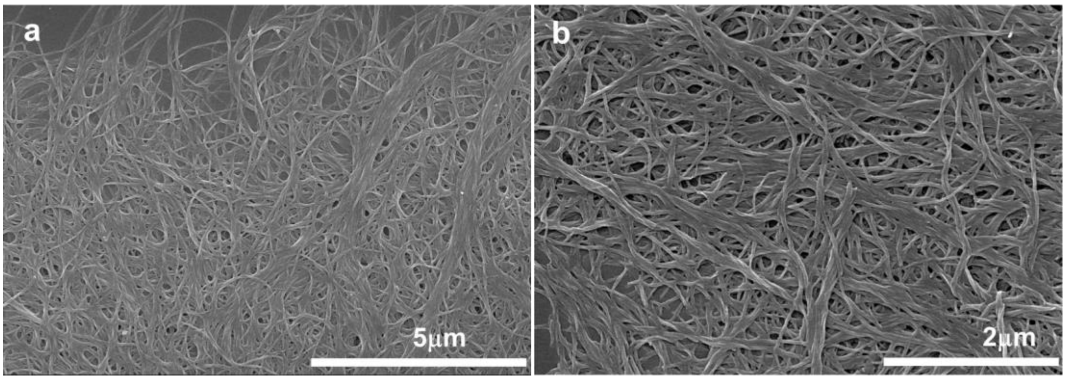

Figure S1: SEM images of NCLG assemblies in EtOH: (a) 2NCLG, (b) 4NCLG. The concentration is 12 mg/mL.

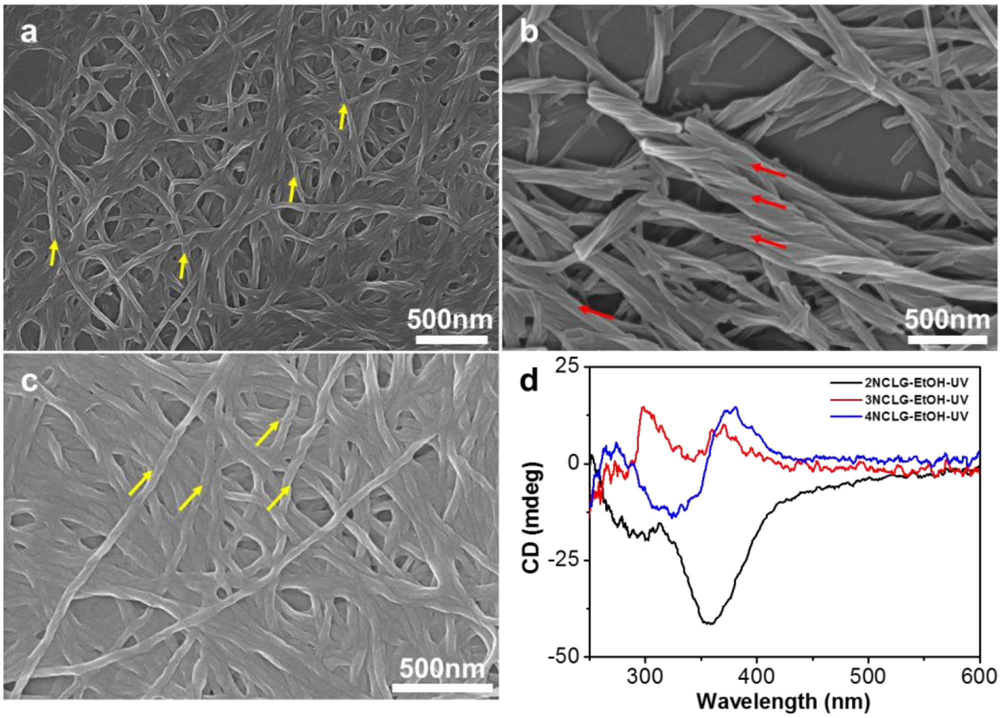

Figure S2: SEM images of NCLG assemblies in EtOH after UV light irradiation: (a) 2NCLG, (b) 3NCLG, (c) 4NCLG. The concentration is 12 mg/mL. (d) CD spectra of NCLG assemblies in EtOH after UV light irradiation.

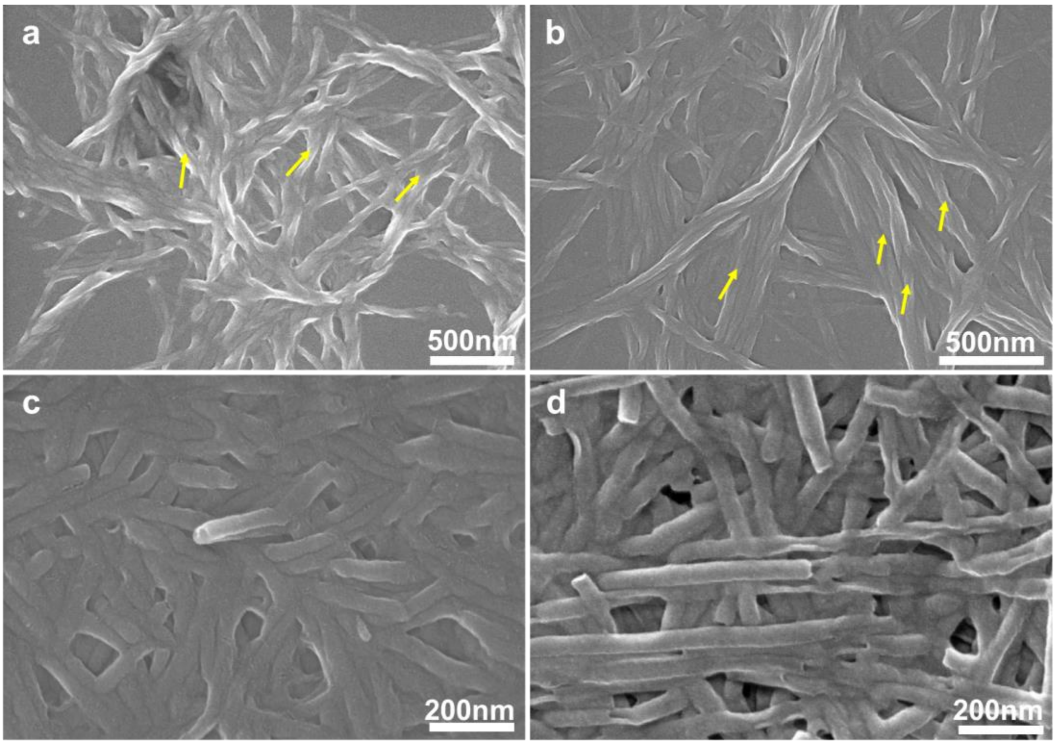

Figure S3: SEM images of 2NCLG assemblies in (a) DMF, (b) THF and 3NCLG assemblies in (c) DMF and (d) THF. The concentration is 12 mg/mL.
